# Genome composition-based deep learning predicts oncogenic potential of HPVs

**DOI:** 10.3389/fcimb.2024.1430424

**Published:** 2024-07-22

**Authors:** Lin Hao, Yu Jiang, Can Zhang, Pengfei Han

**Affiliations:** ^1^ Department of Pharmacy, Linfen Central Hospital, Linfen, China; ^2^ The 4^th^ Medical Center, People's Liberation Army (PLA) General Hospital, Beijing, China

**Keywords:** human papilloma viruses (HPVs), deep learning, oncogenicity, E6, E7

## Abstract

Human papillomaviruses (HPVs) account for more than 30% of cancer cases, with definite identification of the oncogenic role of viral *E6* and *E7* genes. However, the identification of high-risk HPV genotypes has largely relied on lagged biological exploration and clinical observation, with types unclassified and oncogenicity unknown for many HPVs. In the present study, we retrieved and cleaned HPV sequence records with high quality and analyzed their genomic compositional traits of dinucleotide (DNT) and DNT representation (DCR) to overview the distribution difference among various types of HPVs. Then, a deep learning model was built to predict the oncogenic potential of all HPVs based on *E6* and *E7* genes. Our results showed that the main three groups of Alpha, Beta, and Gamma HPVs were clearly separated between/among types in the DCR trait for either *E6* or *E7* coding sequence (CDS) and were clustered within the same group. Moreover, the DCR data of either *E6* or *E7* were learnable with a convolutional neural network (CNN) model. Either CNN classifier predicted accurately the oncogenicity label of high and low oncogenic HPVs. In summary, the compositional traits of HPV oncogenicity-related genes *E6* and *E7* were much different between the high and low oncogenic HPVs, and the compositional trait of the DCR-based deep learning classifier predicted the oncogenic phenotype accurately of HPVs. The trained predictor in this study will facilitate the identification of HPV oncogenicity, particularly for those HPVs without clear genotype or phenotype.

## 1 Introduction

Human papillomaviruses (HPVs) are a group of double-stranded DNA (dsDNA) viruses, specifically tropic to human cutaneous and mucosal epithelial tissues of the anogenital tract, hands, or feet (Correa et al., 2017). There are more than 220 HPV types, which are classified into five genera—Alpha (65 genotypes), Beta (54 genotypes), Gamma (98 genotypes), Mu (four genotypes), and Nu (one genotype) (Muhr et al., 2018) (https://www.hpvcenter.se/)—according to the most genomically homologous viral gene of the major capsid *L1* gene. HPVs exert significant oncogenic roles in various malignant types, unlike most commonly infected viruses, such as adenovirus (Liu et al., 2014), herpes simplex virus (Whitley and Roizman, 2001), influenza viruses (Sun et al., 2014; Deng et al., 2017), and coronaviruses (Sun et al., 2014; Deng et al., 2017). Infectious agents contribute to approximately 12% of global cancers annually (de Martel et al., 2020, 2017). Particularly, HPVs account for more than 30% of cases (https://gco.iarc.fr, accessed on November 15, 2023) (de Martel et al., 2020), such as squamous cell carcinoma (SCC) of the skin (Jablonska et al., 1972), cervical tumors (Walboomers et al., 1999), genital malignancies (de Villiers, 1994), anogenital malignancies (Zur, 2002), and head and neck cancers (Guo et al., 2018). Furthermore, the oncogenic roles of HPVs were heterogenized among various genotypes. HPV16, HPV18, HPV31, HPV33, HPV35, HPV39, HPV45, HPV51, HPV52, HPV56, HPV58, and HPV59 account for the most number of cancers of the cervix, anus, vulva, vagina, penis, and head and neck (Longworth and Laimins, 2004; Galati et al., 2024). HPV16 and HPV18, the most popular high-risk (HR) HPV strains (Bzhalava et al., 2013), account respectively for nearly 50% and 20% of cervical cancer cases (Li and Coffino, 1996; de Sanjose et al., 2010). Thus, it is important to predict and evaluate how an HPV promotes oncogenesis.

There are two main coding regions in the HPV genome (HPV16 as an example): the early (E) region encodes regulatory proteins of E6, E7, E1, E2, E4, and E5; the late (L) region encodes the structural L1 and L2 capsid proteins (Gheit, 2019). Moreover, E6 and E7 are significantly carcinogenic in various types of cancers (Barbosa, 1996; McLaughlin-Drubin and Munger, 2009) by regulating essential cellular progress. E6 protein promotes p53 degradation and thus antagonizes apoptosis (Martinez-Zapien et al., 2016; Shu et al., 2022). E6 also inhibits apoptosis by activating the transcription of survivin and the apoptosis inhibitor c-IAP2 (Bruyere et al., 2023) and regulating host antiviral responses (Yanatatsaneejit et al., 2020). Additionally, HR-HPV E6 binds the protein containing PDZ domains, facilitates its degradation, and thus promotes cell survival and proliferation (Accardi et al., 2011; Drews et al., 2019). The E7 protein also poses oncogenic roles by variously interacting with the DREAM protein complexes (Drosophila, RB, E2F, and Myb) (Rashid et al., 2015) and targeting the pRB family members, which is necessary for malignant transformation (Demers et al., 1996; Zur, 2000; Zhang et al., 2006). Furthermore, HPV E7 proteins exhibit conserved and virus type- and species-specific interactions with cellular proteins (Demers et al., 1996; Zur, 2000; Zhang et al., 2006). Therefore, it is vital to identify the exact association between the genotypes of HPV *E6/E7* and their phenotype for malignant transformation.

Prevalent identification of the association between the genotypes of HPV *E6/E7* and their malignant phenotypes has been recognized to depend on molecular biological and virological approaches (Li and Coffino, 1996; Accardi et al., 2011; White et al., 2012; Martinez-Zapien et al., 2016; Songock et al., 2017; Shu et al., 2022; Bruyere et al., 2023), implying an urgent need for intelligent and fast method to recognize or predict the benign or malignant phenotype of HPVs based on HPV *E6/E7* genotypes. Multiple artificial intelligence (AI) approaches have been utilized to represent genomic information of viruses and to predict viral phenotypes based on such represented information. The deep and hidden viral genotype–phenotype association has been well learned by machine learning (ML) or deep learning (DL) methods. The pandemic potential of influenza viruses (Li et al., 2020), the SARS-CoV-2 adaptation (Li et al., 2020), and the host adaptation of bat coronaviruses (Jiang et al., 2023; Li et al., 2023) have been accurately predicted by ML or DL models. Genomic information of amino acid (Babayan et al., 2018), dinucleotide (DNT) (Li et al., 2020), and DNT representation (DCR) (Li et al., 2022) of the viral genome is predictable and interpretable in the case of genotype–phenotype association. However, the oncogenic phenotype of HPVs analyzed using AI approaches has been less frequently reported.

In this study, we retrieved and cleaned HPV sequence records with high quality and analyzed their genomic traits such as DNT, DCR, and codon dependency. Then, we utilized unsupervised ML approaches to visualize the distribution difference among various types of HPVs. Finally, we built a deep learning model to predict the oncogenic potential of all HPVs based on *E6/E7* genes. The DL model trained in this study can predict the oncogenic potential of any HPV.

## 2 Methods

### 2.1 HPV genome sequence retrieval and data cleaning and the counting of compositional traits of HPV genes

Genome records for HPVs were retrieved using the keywords ((“Human papillomavirus”[Organism] OR Human papillomavirus [All Fields]) AND complete [All Fields] AND genome [All Fields]) AND (biomol_genomic [PROP] AND (“6500” [SLEN]: “8500” [SLEN])) from the National Center for Biotechnology Information (NCBI) nucleotide database (https://www.ncbi.nlm.nih.gov/nuccore). Records were cleaned with sequence quality by excluding the sequence without full coding sequence (CDS) of *E7*, *E6*, *E2*, *E1*, *L2*, and *L1* and the sequence with a ratio of undefined or degenerate bases of more than 2%. The oncogenic label for each sample was manually added according to reported research (Muhr et al., 2018). The compositional traits of DNT and DCR were counted according to the reported tools (Li et al., 2023, 2022). The *E6* and *E7* CDSs were parsed based on the “Location/Qualifiers” information for each record and then were counted under the reported guidance (https://github.com/Jamalijama/BatCoVadaptation). The counted DNT and DCR features were taken as a vector with fixed dimensions of 48 and 1,536. The DCR data were analyzed in detail and utilized for deep learning.

### 2.2 Data distribution analysis with unsupervised machine learning

HPV DCR data were analyzed for the distribution among various HPV types. The dimension reduction was performed using the reported python scripts by the method of t-distributed stochastic neighbor embedding (t-SNE) and principal component analysis (PCA). The 1,536 features of the DCR trait for each sample were reduced into two main components (t-SNE1 and t-SNE2, or PCA1 and PCA2), with packages sklearn.decomposition.PCA and sklearn.manifold.TSNE, both of which were performed according to https://scikit-learn.org/stable/about.html#citing-scikit-learn. The reduced data were plotted for the data distribution difference using the Python Seaborn package of pairplot. Hierarchical clustering was performed to overview the clustering and separation of HPV samples with “type” annotation added into sequence accession. The clustering was calculated using the algorithm of Euclidean distance by the sns.clustermap package.

### 2.3 Building the deep learning model for oncogenic HPV prediction

The deep learning classifier for HPV oncogenicity was built using a model of convolutional neural network (CNN), according to previous reports (Li et al., 2023, 2022). The 1,536-dimensional DCR of each HPV sample (*E6* or *E7*), with an oncogenicity label of 1 or 0, was input into the model. Three rounds of convolution and pooling were performed to learn in-depth the hidden association of DCR with viral oncogenicity phenotype. HPV data were randomly split into training and validating sets using sklearn.model_selection import train_test_split with a validation data size of 25%. The 1,536-dimensional DCR was first reshaped into a matrix of 6 × 16 × 16 for a 3D-CNN performance. Out-channels were set as 8, 16, and 32. A kernel size of (1, 3, 3), a rectified linear unit (ReLU) activation, and the average pooling were utilized for each CNN layer. Two linear transformations were performed to transform the 768-dimensional vector first into the 192-dimensional vector and then into the two-dimensional final predicted labels of 1 and 0. The Softmax function was utilized to output the prediction probability. A learning rate of 0.01 and a training epoch of 50 were utilized for either model.

To evaluate the prediction performance of each classifier, micro-average receiver operating characteristic (ROC) (Fawcett, 2005) and confusion matrix (Fawcett, 2005) were plotted. The full-connection data after the three rounds of convolution performance were reduced by PCA and then were plotted for the two main components.

### 2.4 Statistics

Statistical analysis was performed for the significance in the PCA1 value of the PCA-reduced full-connection data and analyzed using an unpaired, non-parametric Mann–Whitney test using the SciPy scripts of python.

## 3 Results

### 3.1 Pipeline of genomic trait analysis and oncogenic potential prediction of HPVs

**Figure 1:**
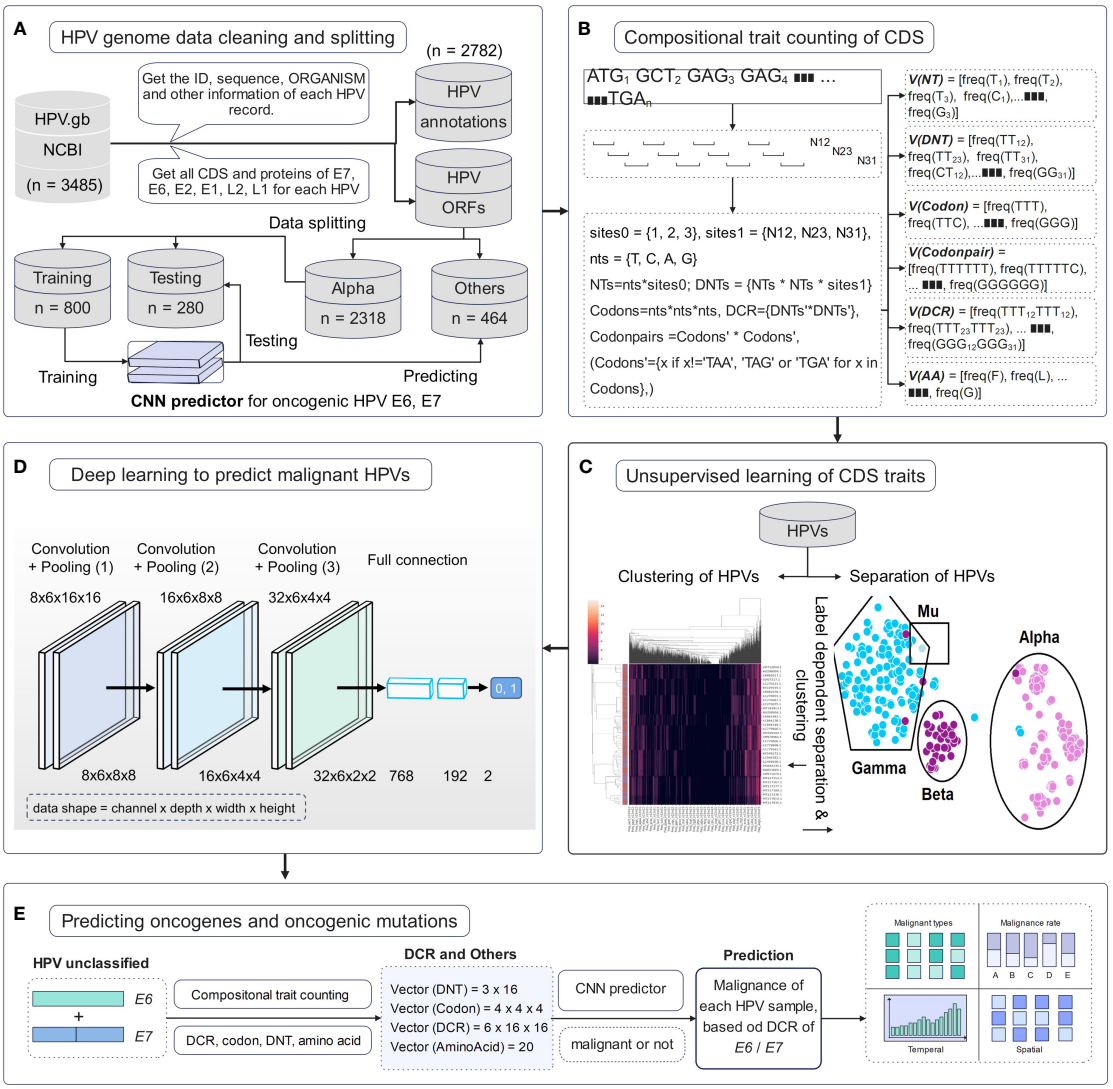
Pipeline of data cleaning and deep learning analysis of human papillomavirus (HPV) genes. **(A)** HPV data cleaning process and the sequence number for the HPVs with full *E6* and *E7* genes. **(B)** Illustration of counting method for the compositional traits of HPV *E6* and *E7* genes. **(C)** Illustration of dimension reduction and unsupervised learning of HPV genes. **(D)** The structure of convolutional neural network (CNN) model and the detailed dimension of dinucleotide representation (DCR) data during CNN. **(E)** The prediction of the oncogenicity of HPVs by the CNN classifier and the analysis of oncogenic HPVs.

Full HPV genome information with genomic DNA sequences and annotations was downloaded from the NCBI website. The 3,485 HPV records were cleaned to obtain high-quality samples. A total of 2,782 samples were parsed to obtain the annotation set of record ID, ORGANISM, and other information and then to obtain all CDS and protein sequences. Data were separated according to HPV type, and then Alpha HPVs with definite high or low oncogenicity were randomly split into training and testing sets. All other HPVs, particularly unclassified HPVs, were predicted using a trained deep learning predictor (**Figure 1A**). Genomic compositional traits of HPV *E6* and *E7* CDSs were counted according to the reported method (Li et al., 2023, 2022) (**Figure 1B**). Unsupervised ML approaches were performed to analyze the distribution difference of HPV genomic traits among various types. Features of DNT, codon, DCR, and others were first reduced using the method of PCA or t-SNE and then plotted for the two main components. Full feature data for each type of trait were clustered using a hierarchical clustering method (**Figure 1C**). A deep learning network of CNN was trained with the DCR trait data of HPV *E6* or *E7* for oncogenicity prediction of HPVs (**Figure 1D**). Finally, each HPV sample was predicted using a trained model, and the temporal/spatial distribution of HPVs with predicted high or low oncogenicity was analyzed (**Figure 1E**).

### 3.2 Data distribution of HPVs and type-based clustering and separation of HPVs revealed by unsupervised ML methods

**Figure 2:**
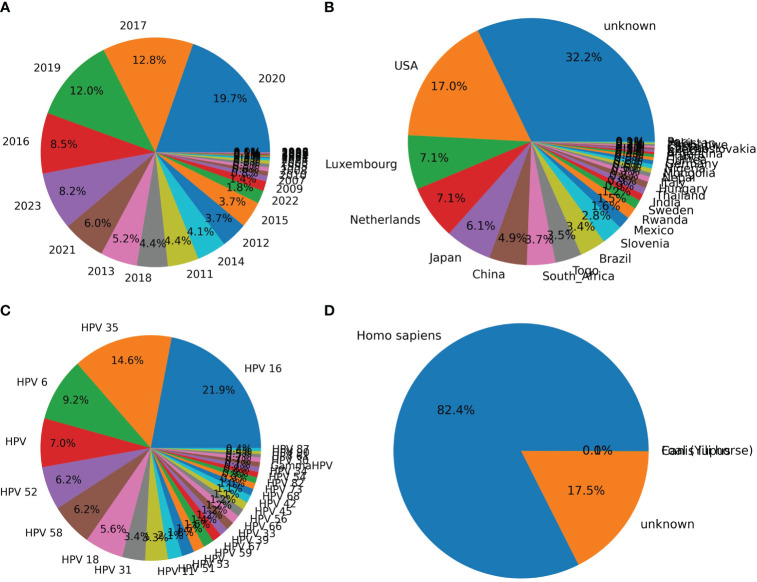
Counting of annotation items for human papillomaviruses (HPVs) based on original recorded information. The counting of HPVs based on labels “year” **(A)**, “country” **(B)**, “genotype” **(C)**, and “host” **(D)** was performed based on the above-mentioned annotation for each HPV record on National Center for Biotechnology Information (NCBI) website.

The distribution of HPVs on the annotation of date, country, genotype, and isolation host was counted based on the parsed HPV data. Most of the recorded HPVs on BCBI were isolated post-2010 (**Figure 2A**). More than one-third of the samples were not annotated for their isolation country, and most of the annotated samples were from the USA, Luxembourg and the Netherlands (the EU), Japan and China (East Asia), and South Africa (**Figure 2B**). Approximately 1/10 HPV samples were not annotated for their isolation hosts, and the top genotypes were HPV16, HPV35, HPV52, HPV58, HPV18, HPV31, HPV11, HPV51, and HPV53, which are high or low oncogenic HPVs (**Figure 2C**). Additionally, the isolation annotation was also not clear for more than one-fifth of the samples (**Figure 2D**). Thus, it is important to build an intelligent predictor for identifying the oncogenicity of these HPVs with unknown annotations.

**Figure 3:**
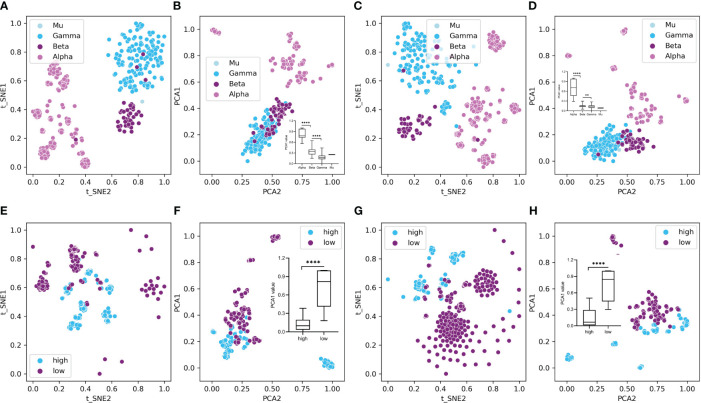
Plot of dinucleotide representation (DCR) data of the human papillomaviruses (HPVs), with type label or oncogenicity label. The DCR data with 1,536 dimensions were first reduced with t-distributed stochastic neighbor embedding (t-SNE) and principal component analysis (PCA) and then were plotted with the two main reduced components, with type or oncogenicity labeled for each sample plot. **(A–D)** t-SNE- and PCA-reduced DCR for *E6* **(A, B)** or *E7* gene **(C, D)** of HPVs, with “type” labeled. **(E–H)** t-SNE- and PCA-reduced DCR for *E6* **(E, F)** or *E7* gene **(G, H)** of HPVs, with “oncogenicity” labeled.

To overview the HPV distribution among the four types (Alpha, Beta, Gamma, and Mu) of HPVs or all genotypes, full data of compositional DCR or other traits were reduced into two main components with t-SNE or PCA and then plotted with type information annotated for each sample. As indicated, the main three groups (Alpha, Beta, Gamma, only two samples for Mu HPVs) were clearly separated in DCR trait for *E6* CDS (**Figure 3A** by t-SNE and **Figure 3B** by PCA) or *E7* CDS (**Figure 3C** by t-SNE and **Figure 3D** by PCA). Moreover, the Alpha HPVs were further analyzed using the same methods to visualize the distribution variation between high and low oncogenic HPVs. Both *E6* CDS (**Figure 3E** by t-SNE and **Figure 3F** by PCA) and *E7* CDS sequence (**Figure 3G** by t-SNE and **Figure 3H** by PCA) were clearly separated.

**Figure 4:**
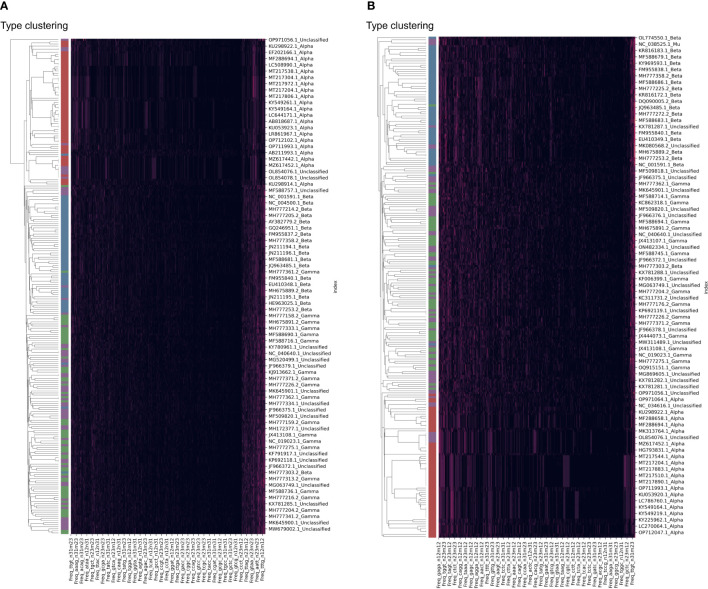
Clustering of human papillomavirus (HPV) samples based on full dinucleotide representation (DCR) data, with viral type visualized. HPVs were randomly sampled and added with type labels into the accession number. The DCR data were hierarchically clustered based on the Euclidean distance within these samples for *E6* **(A)** and *E7* **(B)** genes.

The clustering of HPV samples was also performed based on the DCR trait. The HPV clustering into the main three groups of Alpha, Beta, and Gamma was interrupted by unclassified samples based on either the *E6* (**Figure 4A**) or *E7* (**Figure 4B**) gene. Therefore, it is necessary to identify the classification of these unannotated samples.

### 3.3 Deep learning model predicted the oncogenic potential of HPVs based on viral genomic composition of *E6* or *E7*

**Figure 5:**
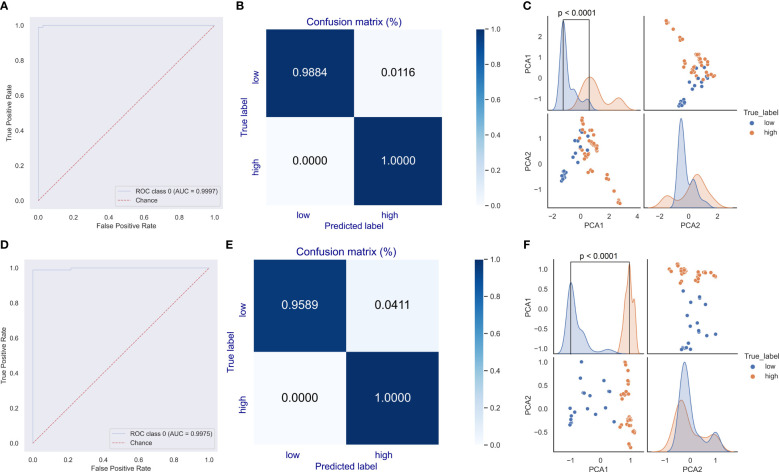
Evaluation of the convolutional neural network (CNN) predictor for human papillomavirus (HPV) oncogenicity. The trained CNN predictors were evaluated for their classification performance with receiver operating characteristic (ROC) **(A)** and confusion matrix **(B)** for *E6* gene. The distribution difference in the full-connection dinucleotide representation (DCR) data between low and high oncogenic HPVs was plotted using a pairplot method **(C)**. Similar ROC, confusion matrix, and full-connection DCR data are plotted **(D–F)**.

Given the dominant roles of *E6* and *E7* on the oncogenicity of HPVs, a deep learning predictor of the CNN model was built for the prediction of unclassified HPVs. The network structure is illustrated in **Figure 1D**. The DCR vector of 1,536 dimensions was sequentially convoluted and pooled three times, and then the outputted full-connection vector of 768 dimensions was linearly transformed into a vector of 192 dimensions, with final two oncogenicity labels of 1 (oncogenicity positive) and 0 (oncogenicity negative). The DCR-based CNN classification for either *E6* or *E7* was trained. The predictor for *E6* was highly accurate for the independent HPV test data based on the ROC_AUC curve (**Figure 5A**); after a training epoch of 50, the accuracy was indicated by the confusion matrix (**Figure 5B**). The full-connection data of *E6* DCR were clearly separated based on the distribution of the two main PCA-reduced components. The PCA1 peaks of high and low oncogenic HPVs were significantly different (p < 0.0001, **Figure 5C**). The performance of the *E7* CNN predictor was also highly accurate. The ROC_AUC curve was almost at a right angle (**Figure 5D**), and the accuracy was more than 95% for low oncogenic HPVs and almost 100% for high oncogenic HPVs (**Figure 5E**). Similarly, the difference between the two PCA1 peaks was very significant (p < 0.0001, **Figure 5F**). Taken together, both *E6* and *E7* genes were greatly but vaguely different and learnable with the deep learning method.

### 3.4 Deep learning prediction of unclassified HPVs of the oncogenic potential based on *E6* or *E7*

**Figure 6:**
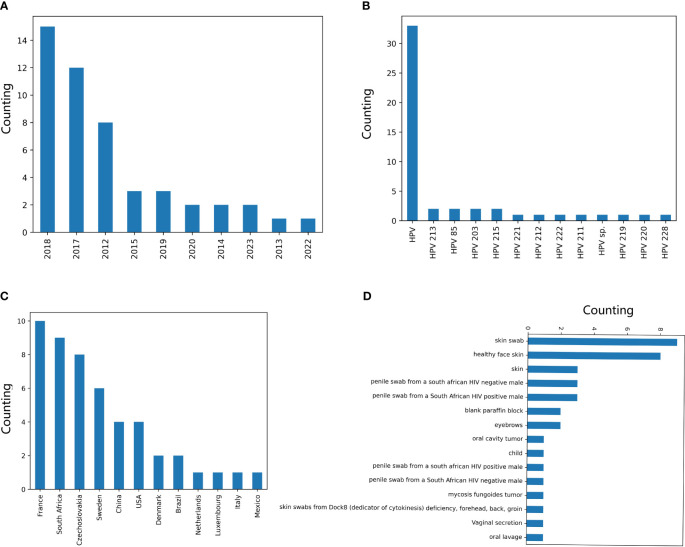
Counting of annotation items for the human papillomaviruses (HPVs), with oncogenicity predicted. The counting of the HPVs, with the oncogenicity predicted using the CNN classifier based on *E7* dinucleotide representation (DCR) for labels “year” **(A)**, “genotype” **(B)**, “country” **(C)**, and “isolation_source” **(D)**.

The two CNN predictors based on DCR data of *E6* and *E7* were utilized to predict the oncogenicity of HPVs. The 67 unclassified HPVs with full CDS of *E6* and *E7* were first counted for the DCR trait and then were input into the trained CNN predictor for either *E6* or *E7*. The predicted label of 1 or 0, the probability for each label, and the full annotation information are listed in **Supplementary Table 1**. As indicated, 49/67 samples were predicted to be oncogenic based on the *E6* DCR trait, whereas 9/67 unclassified HPVs were predicted to be oncogenic based on the *E7* DCR trait. The distribution of data, genotype, country, and collection site was plotted. The 49 positive samples predicted by *E7* DCR were mainly isolated in 2018, 2017, and 2012 (**Figure 6A**), mostly without annotated genotype (**Figure 6B**), isolated in EU countries (**Figure 6C**), and mainly collected from skin and penile swab samples (**Figure 6D**). Taken together, the present deep learning predictors facilitated the oncogenicity evaluation based on viral *E6* and *E7* genes.

## 4 Discussion

In response to the need to evaluate the oncogenic phenotype of HPVs, particularly for the novel HPV strains, without identified genotypes, or the HPV with its oncogenicity unclear, we utilized the reported method to parse the genomic compositional traits of HPV CDSs, and trained deep learning classifiers, based on HPV *E6* or *E7* DCR data. For most types of viruses, the genome sequences do not have the same length and need to be aligned for sequence similarity analysis using Multiple Sequence Alignment (MSA). However, the high computational consumption and complexity were not balanced with an intelligent evaluation of aligned sequences. The compositional DNT, DCR, and other codon-dependent traits of CDSs (Li et al., 2022, 2020) were MSA-independent and have fixed dimensions, which make further analysis easy. More importantly, such compositional traits were more biologically interpretable for virus prediction (Jiang et al., 2023; Li et al., 2023, 2022). Thus, the compositional trait of DCR with fixed dimension is more applicable to represent the genotype difference for viral genes with various sequence lengths, such as HPVs.

The oncogenicity of HPVs has been explored using AI approaches. Most of these explorations focused on diagnostic image recognition (Fu et al., 2022; La Greca et al., 2022; Klein et al., 2023; de Sanjose et al., 2024). Rare reports implicated the oncogenicity assessment of HPVs based on their genotype polymorphism. However, more and more HPV genome samples have been isolated and sequenced, and there are more novel HPV genotypes that have been identified in recent years. However, the biological identification and clinical observation of the oncogenicity of these HPV strains were much more lagged. Interestingly, the trained predictor in this study is capable of predicting the oncogenicity of these unknown genotypes, such as HPV211–215. Thus, the compositional trait-based deep learning classifiers have provided alternative and intelligent tools for HPV evaluation.

The present study analyzed the compositional characteristics of HPV *E6* and *E7* genes, which are most important for the virus oncogenicity for the first time. Our results have clearly illustrated a significant type- or genotype-dependent clustering or separation of HPVs, particularly for the high oncogenic and benign HPVs. Such marked difference in DCR distribution between the two groups was easily learned by a CNN model for the classification of the two virus groups. The high accuracy of our trained predictor on independent test data implied that the two models were reliable for the oncogenicity evaluation of HPVs. Interestingly, a much more significant difference in the DCR trait between high and low oncogenic HPVs was indicated by the PCA-reduced DCR data, implying more significance on the viral oncogenicity. The supervised learning of the CNN classifier predicted many more HPVs without clear oncogenicity, and genotyping was predicted to be oncogenic based on *E7* CDS than based on *E6*. The dominant promotion by HPV *E6* and *E7* genes to the malignant transformation of viral targeted cells has been biologically explored and widely clinically studied for a long time. However, the comparable importance between *E6* and *E7* genes is not clear. The significant difference in the oncogenicity prediction by *E6*- and *E7*-based classifiers implied different importance of the two genes.

Additionally, other HPV genes like *E2*, *E1*, *L2*, and *L1* have been indicated to be less associated with the oncogenicity of HPVs. However, their biological roles in the HPV–host interaction have less been focused, compared to *E6* and *E7*. The present study might also be beneficial for the analysis of the phenotype–genotype relationship for these genes. However, such analysis of prediction for the topic of oncogenicity of HPVs should be more feasible owing to the knowledge of the association between viral oncogenicity and the genotypes in these genes.

## 5 Conclusion

In summary, the viral genomic compositional traits characterize the oncogenicity of HPVs and facilitate the prediction of the high or low oncogenic HPVs via a DCR-based deep learning classifier for the oncogenicity-related genes *E6* and *E7*. The trained predictor in this study will facilitate the identification of HPV oncogenicity, particularly for those HPVs without clear genotype or phenotype.

## Data availability statement

The original contributions presented in the study are included in the article/**Supplementary Material**. Further inquiries can be directed to the corresponding author.

## Author contributions

LH: Conceptualization, Formal analysis, Methodology, Project administration, Resources, Software, Supervision, Writing – original draft, Writing – review & editing, Data curation, Visualization, Funding acquisition, Investigation, Validation. YJ: Methodology, Software, Writing – original draft, Investigation, Resources. CZ: Investigation, Methodology, Software, Writing – original draft, Visualization. PH: Investigation, Methodology, Software, Visualization, Writing – original draft, Conceptualization, Formal analysis, Project administration, Resources, Supervision, Writing – review & editing, Data curation, Funding acquisition, Validation.
