# Supplementary material for: Genome composition-based deep learning predicts oncogenic potential of HPVs
Source: Front Cell Infect Microbiol. 2024 Jul 22;14:1430424. doi: 10.3389/fcimb.2024.1430424 (PMC11298479; doi:10.3389/fcimb.2024.1430424)
Supplement: Supplementary file 1 [file Table_1.docx]

**Supplementary Table 1 Oncogenicity prediction of unclassified HPVs based on genomic DCR with full CDS information of E6 and E7.**

| seqID | seqlen | | | date | | | year | | | source | | | type | | | organism | | | collect_date | | country | | | host | | | isolate | | | isolation_source | | | Accession_E7 | | pred_label_E7 | | Score_0_E7 | | | Score_1_E7 | | Accession_E6 | | | pred_label_E6 | | | Score_0_E6 | | | Score_1_E6 | | |
| --- | --- | --- | --- | --- | --- | --- | --- | --- | --- | --- | --- | --- | --- | --- | --- | --- | --- | --- | --- | --- | --- | --- | --- | --- | --- | --- | --- | --- | --- | --- | --- | --- | --- | --- | --- | --- | --- | --- | --- | --- | --- | --- | --- | --- | --- | --- | --- | --- | --- | --- | --- | --- | --- |
| MF176072.1 | 7216 | | | 2017-10-16 | | | 2017 | | | Human papillomavirus | | | Unclassified | | | HPV | | | ####### | | China | | | Homo sapiens | | | TG550 | | |  | | | MF176072.1 | | 1 | | 6.12E-10 | | | 1 | | MF176072.1 | | | 0 | | | 1 | | | 4.54E-08 | | |
| KY349817.1 | 7405 | | | 2017-09-21 | | | 2017 | | | Human papillomavirus | | | Unclassified | | | HPV | | | 28-Jul-14 | | USA | | | Homo sapiens | | | MTS1 | | | skin | | | KY349817.1 | | 1 | | 8.59E-11 | | | 1 | | KY349817.1 | | | 1 | | | 0.059663 | | | 0.940338 | | |
| KX781281.1 | | 7330 | | | 2017-02-15 | | | 2017 | | | Human papillomavirus | | | Unclassified | | | 16-Jul | | | Czechoslovakia | | | Dysk2 | | | skin swab | | | KX781281.1 | | | 1 | | | 2.80E-07 | | | 1 | | | KX781281.1 | | | 0 | | | 1 | | | 1.95E-07 | | |  |
| MF509818.1 | 7096 | | | 2018-04-13 | | | 2018 | | | Human papillomavirus type 213 | | | Unclassified | | | HPV 213 | | | 2007 | | South Africa | | | Homo sapiens | | | CT04_15 | | | penile swab from a south african HIV negative male | | | MF509818.1 | | 1 | | 1.60E-07 | | | 1 | | MF509818.1 | | | 0 | | | 0.996412 | | | 0.003588 | | |
| MG869605.1 | | | 7240 | | | 2018-09-10 | | | 2018 | | | Human papillomavirus | | | Unclassified | | | Denmark | | | | Homo sapiens | | | oral cavity tumor | | | MG869605.1 | | | 1 | | | 0.066173 | | 0.933827 | | | MG869605.1 | | | | 0 | | | 0.995088 | | | 0.004912 | | |  |  |
| MH172378.1 | 7326 | | | 2018-05-20 | | | 2018 | | | Human papillomavirus type 221 | | | Unclassified | | | HPV 221 | | | 2007 | | South Africa | | | Homo sapiens | | | CT09 | | | penile swab from a South African HIV positive male | | | MH172378.1 | | 1 | | 4.27E-10 | | | 1 | | MH172378.1 | | | 0 | | | 0.999975 | | | 2.52E-05 | | |
| JF966375.1 | 7286 | | | 2012-01-11 | | | 2012 | | | Human papillomavirus | | | Unclassified | | | HPV | | | 9-May | | France | | | Homo sapiens | | | 915 F 06 007 FD1 | | | healthy face skin | | | JF966375.1 | | 0 | | 0.999958 | | | 4.20E-05 | | JF966375.1 | | | 0 | | | 1 | | | 1.44E-11 | | |
| KC113191.1 | | 7299 | | | 2013-04-04 | | | 2013 | | | Human papillomavirus | | | Unclassified | | | 2008 | | | USA | | | Homo sapiens | | | HPV_SD2R.align1 | | | KC113191.1 | | | 0 | | | 0.999302 | | | 0.000698 | | | KC113191.1 | | | 0 | | | 0.999991 | | | 8.75E-06 | | |  |
| KC311731.2 | | 7247 | | | 2013-09-03 | | | 2013 | | | Human papillomavirus | | | Unclassified | | | 2008 | | | Finland | | | Homo sapiens | | | nasal swab | | | KC311731.2 | | | 0 | | | 0.99618 | | | 0.00382 | | | KC311731.2 | | | 0 | | | 1 | | | 4.51E-09 | | |  |
| JF966371.1 | 7300 | | | 2012-01-11 | | | 2012 | | | Human papillomavirus | | | Unclassified | | | HPV | | | 9-May | | France | | | Homo sapiens | | | 915 F 06 002 KN1 | | | healthy face skin | | | JF966371.1 | | 1 | | 0.00023 | | | 0.99977 | | JF966371.1 | | | 0 | | | 1 | | | 5.64E-09 | | |
| MF356498.1 | 7233 | | | 2017-09-21 | | | 2017 | | | Human papillomavirus | | | Unclassified | | | HPV | | | 28-Jul-14 | | USA | | | Homo sapiens | | | ICB1 | | | skin | | | MF356498.1 | | 1 | | 3.98E-07 | | | 1 | | MF356498.1 | | | 0 | | | 0.999996 | | | 3.82E-06 | | |
| JF966372.1 | 7299 | | | 2012-01-11 | | | 2012 | | | Human papillomavirus | | | Unclassified | | | HPV | | | 9-May | | France | | | Homo sapiens | | | 915 F 06 002 KN2 | | | healthy face skin | | | JF966372.1 | | 1 | | 0.290397 | | | 0.709603 | | JF966372.1 | | | 0 | | | 1 | | | 6.16E-12 | | |
| OL854080.1 | 7994 | | | 2022-11-15 | | | 2022 | | | Human papillomavirus | | | Unclassified | | | HPV | | | 1-Nov-21 | | China: ShanDong, Jinan | | | Homo sapiens | | | 1098 | | | pathological tissue | | | OL854080.1 | | 0 | | 1 | | | 5.93E-13 | | OL854080.1 | | | 0 | | | 1 | | | 2.30E-08 | | |
| MK080568.2 | 7441 | | | 2020-02-11 | | | 2020 | | | Human papillomavirus | | | Unclassified | | | HPV | | | ####### | | USA | | | Homo sapiens | | | ICB2 | | | skin | | | MK080568.2 | | 1 | | 5.51E-06 | | | 0.999995 | | MK080568.2 | | | 0 | | | 0.999987 | | | 1.26E-05 | | |
| NC_034616.1 | | 7812 | | | 2018-08-25 | | | 2018 | | | Human papillomavirus type 85 | | | Unclassified | | | Brazil | | | Homo sapiens | | | 114B | | |  | | | NC_034616.1 | | | 1 | | | 0.00098 | | | 0.99902 | | | NC_034616.1 | | | 1 | | | 4.56E-11 | | | 1 | | |  |
| MK645901.1 | 7283 | | | 2019-07-08 | | | 2019 | | | Human papillomavirus | | | Unclassified | | | HPV | | | ####### | | Spain | | | Homo sapiens | | | type 208 | | | oral mucosa | | | MK645901.1 | | 0 | | 0.989719 | | | 0.010281 | | MK645901.1 | | | 0 | | | 1 | | | 3.67E-11 | | |
| KF482069.1 | 7177 | | | 2013-10-24 | | | 2013 | | | Human papillomavirus | | | Unclassified | | | HPV | | | 2013 | | China | | | Homo sapiens | | | HPV-L55 | | | child | | | KF482069.1 | | 1 | | 0.143276 | | | 0.856724 | | KF482069.1 | | | 1 | | | 0.000368 | | | 0.999632 | | |
| MK645900.1 | 7247 | | | 2019-07-08 | | | 2019 | | | Human papillomavirus | | | Unclassified | | | HPV | | | ####### | | Spain | | | Homo sapiens | | | type 207 | | | oral mucosa | | | MK645900.1 | | 0 | | 0.909942 | | | 0.090058 | | MK645900.1 | | | 0 | | | 0.9133 | | | 0.0867 | | |
| KP692119.1 | 7292 | | | 2015-08-19 | | | 2015 | | | Human papillomavirus | | | Unclassified | | | HPV | | | 2014 | | Sweden | | | Homo sapiens | | | SE435 | | |  | | | KP692119.1 | | 1 | | 4.18E-12 | | | 1 | | KP692119.1 | | | 0 | | | 0.999999 | | | 1.17E-06 | | |
| KX781284.1 | | 7281 | | | 2017-02-15 | | | 2017 | | | Human papillomavirus | | | Unclassified | | | 16-Jul | | | Czechoslovakia | | | Dysk5 | | | skin swab | | | KX781284.1 | | | 1 | | | 0.190101 | | | 0.809899 | | | KX781284.1 | | | 0 | | | 1 | | | 2.43E-16 | | |  |
| KX781287.1 | | 7375 | | | 2017-02-15 | | | 2017 | | | Human papillomavirus | | | Unclassified | | | 16-Jul | | | Czechoslovakia | | | DyskC | | | skin swab | | | KX781287.1 | | | 1 | | | 3.26E-09 | | | 1 | | | KX781287.1 | | | 1 | | | 0.347672 | | | 0.652328 | | |  |
| OP971056.1 | 7357 | | | 2023-01-14 | | | 2023 | | | human papillomavirus 214 | | | Unclassified | | | HPV 214 | | | 2-Mar-17 | | South Africa | | | Homo sapiens | | | UC113_LW_V2 | | | Vaginal secretion | | | OP971056.1 | | 0 | | 0.984311 | | | 0.015689 | | OP971056.1 | | | 0 | | | 1 | | | 4.96E-17 | | |
| KJ194499.1 | | 7705 | | | 2014-04-08 | | | 2014 | | | Human papillomavirus | | | Unclassified | | | ####### | | | Netherlands | | | Homo sapiens | | | HPV_Amsterdam_1995 | | | KJ194499.1 | | | 1 | | | 8.76E-12 | | | 1 | | | KJ194499.1 | | | 1 | | | 6.02E-09 | | | 1 | | |  |
| KX514426.1 | | 7812 | | | 2017-05-18 | | | 2017 | | | Human papillomavirus type 85 | | | Unclassified | | | Brazil | | | Homo sapiens | | | 114B | | |  | | | KX514426.1 | | | 1 | | | 0.00098 | | | 0.99902 | | | KX514426.1 | | | 1 | | | 4.56E-11 | | | 1 | | |  |
| KP692118.1 | 7317 | | | 2015-08-19 | | | 2015 | | | Human papillomavirus | | | Unclassified | | | HPV | | | 2014 | | Sweden | | | Homo sapiens | | | SE383 | | |  | | | KP692118.1 | | 1 | | 4.18E-12 | | | 1 | | KP692118.1 | | | 0 | | | 0.999999 | | | 1.17E-06 | | |
| NC_040640.1 | 7372 | | | 2019-09-12 | | | 2019 | | | Human papillomavirus type 203 | | | Unclassified | | | HPV 203 | | | 2014 | | Sweden | | |  | | | SE369 | | | blank paraffin block | | | NC_040640.1 | | 1 | | 0.285294 | | | 0.714706 | | NC_040640.1 | | | 0 | | | 1 | | | 4.32E-09 | | |
| MF509820.1 | 7186 | | | 2018-04-13 | | | 2018 | | | Human papillomavirus type 215 | | | Unclassified | | | HPV 215 | | | 2007 | | South Africa | | | Homo sapiens | | | CT07_2 | | | penile swab from a south african HIV negative male | | | MF509820.1 | | 1 | | 1.28E-11 | | | 1 | | MF509820.1 | | | 0 | | | 1 | | | 2.85E-07 | | |
| MF509817.1 | 7208 | | | 2018-04-13 | | | 2018 | | | Human papillomavirus type 212 | | | Unclassified | | | HPV 212 | | | 2007 | | South Africa | | | Homo sapiens | | | CT03_1 | | | penile swab from a south african HIV positive male | | | MF509817.1 | | 1 | | 0.001683 | | | 0.998317 | | MF509817.1 | | | 0 | | | 0.997821 | | | 0.002179 | | |
| MG921180.1 | 7372 | | | 2018-06-05 | | | 2018 | | | Human papillomavirus type 203 | | | Unclassified | | | HPV 203 | | | 2014 | | Sweden | | |  | | | SE369 | | | blank paraffin block | | | MG921180.1 | | 1 | | 0.285294 | | | 0.714706 | | MG921180.1 | | | 0 | | | 1 | | | 4.32E-09 | | |
| MH172379.1 | 7275 | | | 2018-05-20 | | | 2018 | | | Human papillomavirus type 222 | | | Unclassified | | | HPV 222 | | | 2007 | | South Africa | | | Homo sapiens | | | CT17 | | | penile swab from a South African HIV negative male | | | MH172379.1 | | 1 | | 1.22E-12 | | | 1 | | MH172379.1 | | | 0 | | | 1 | | | 3.36E-10 | | |
| MG520499.1 | 7320 | | | 2018-03-21 | | | 2018 | | | Human papillomavirus | | | Unclassified | | | HPV | | | 1-Jan-17 | | France | | | Homo sapiens | | | MTS4 | | | eyebrows | | | MG520499.1 | | 1 | | 0.000108 | | | 0.999892 | | MG520499.1 | | | 0 | | | 1 | | | 3.55E-09 | | |
| OL854078.1 | 7994 | | | 2022-11-15 | | | 2022 | | | Human papillomavirus | | | Unclassified | | | HPV | | | 1-Nov-21 | | China: ShanDong, Jinan | | | Homo sapiens | | | 1122 | | | pathological tissue | | | OL854078.1 | | 0 | | 1 | | | 5.93E-13 | | OL854078.1 | | | 0 | | | 1 | | | 2.30E-08 | | |
| OL854079.1 | 7994 | | | 2022-11-15 | | | 2022 | | | Human papillomavirus | | | Unclassified | | | HPV | | | 1-Nov-21 | | China: ShanDong, Jinan | | | Homo sapiens | | | 723 | | | pathological tissue | | | OL854079.1 | | 0 | | 1 | | | 5.93E-13 | | OL854079.1 | | | 0 | | | 1 | | | 2.30E-08 | | |
| MF509816.1 | 7253 | | | 2018-04-13 | | | 2018 | | | Human papillomavirus type 211 | | | Unclassified | | | HPV 211 | | | 2007 | | South Africa | | | Homo sapiens | | | CT02_8 | | | penile swab from a south african HIV negative male | | | MF509816.1 | | 1 | | 4.27E-09 | | | 1 | | MF509816.1 | | | 0 | | | 0.999946 | | | 5.36E-05 | | |
| OL854076.1 | 7994 | | | 2022-11-15 | | | 2022 | | | Human papillomavirus | | | Unclassified | | | HPV | | | 1-Nov-21 | | China: ShanDong, Jinan | | | Homo sapiens | | | 2004 | | | pathological tissue | | | OL854076.1 | | 0 | | 1 | | | 5.93E-13 | | OL854076.1 | | | 0 | | | 1 | | | 2.30E-08 | | |
| OL854077.1 | 7994 | | | 2022-11-15 | | | 2022 | | | Human papillomavirus | | | Unclassified | | | HPV | | | 1-Nov-21 | | China: ShanDong, Jinan | | | Homo sapiens | | | 1162 | | | pathological tissue | | | OL854077.1 | | 0 | | 1 | | | 5.93E-13 | | OL854077.1 | | | 0 | | | 1 | | | 2.30E-08 | | |
| LR862055.1 | | | 7096 | | | 2020-09-01 | | | 2020 | | | Human papillomavirus type 213 | | | Unclassified | | | Luxembourg | | | | Homo sapiens | | | LNS8643648_HPV213 | | | LR862055.1 | | | 1 | | | 1.60E-07 | | 1 | | | LR862055.1 | | | | 0 | | | 0.997563 | | | 0.002437 | | |  |  |
| MG869604.1 | | 7291 | | | 2018-09-10 | | | 2018 | | | Human papillomavirus | | | Unclassified | | | ####### | | | Denmark | | | Homo sapiens | | | mycosis fungoides tumor | | | MG869604.1 | | | 1 | | | 5.83E-06 | | | 0.999994 | | | MG869604.1 | | | 0 | | | 0.999929 | | | 7.15E-05 | | |  |
| KX781283.1 | | 7306 | | | 2017-02-15 | | | 2017 | | | Human papillomavirus | | | Unclassified | | | 16-Jul | | | Czechoslovakia | | | Dysk4 | | | skin swab | | | KX781283.1 | | | 1 | | | 0.001862 | | | 0.998138 | | | KX781283.1 | | | 0 | | | 1 | | | 2.55E-07 | | |  |
| JF966374.1 | 7251 | | | 2012-01-11 | | | 2012 | | | Human papillomavirus | | | Unclassified | | | HPV | | | 9-May | | France | | | Homo sapiens | | | 915 F 06 002 KN3 | | | healthy face skin | | | JF966374.1 | | 1 | | 0.017846 | | | 0.982155 | | JF966374.1 | | | 0 | | | 1 | | | 2.62E-16 | | |
| KY780961.1 | 7319 | | | 2017-09-21 | | | 2017 | | | Human papillomavirus | | | Unclassified | | | HPV | | | 29-Jul-14 | | USA | | | Homo sapiens | | | MTS2 | | | skin | | | KY780961.1 | | 0 | | 0.814786 | | | 0.185214 | | KY780961.1 | | | 0 | | | 1 | | | 7.91E-08 | | |
| NC_027779.1 | 7320 | | | 2018-08-13 | | | 2018 | | | Human papillomavirus | | | Unclassified | | | HPV | | | 2014 | | Sweden | | | Homo sapiens | | | SE379 | | |  | | | NC_027779.1 | | 1 | | 7.65E-05 | | | 0.999923 | | NC_027779.1 | | | 0 | | | 0.868107 | | | 0.131893 | | |
| MF588757.1 | 7203 | | | 2019-03-22 | | | 2019 | | | Human papillomavirus sp | | | Unclassified | | | HPV sp. | | | 14-May | | Italy | | | Homo sapiens | | | Mu3_d01c06 | | | skin swabs from Dock8 (dedicator of cytokinesis) deficiency, forehead, back, groin | | | MF588757.1 | | 1 | | 0.01065 | | | 0.98935 | | MF588757.1 | | | 0 | | | 0.933794 | | | 0.066206 | | |
| MH172376.1 | 7108 | | | 2018-05-20 | | | 2018 | | | Human papillomavirus type 219 | | | Unclassified | | | HPV 219 | | | 2007 | | South Africa | | | Homo sapiens | | | CT01 | | | penile swab from a South African HIV positive male | | | MH172376.1 | | 1 | | 0.000526 | | | 0.999474 | | MH172376.1 | | | 0 | | | 0.999512 | | | 0.000488 | | |
| KX781285.1 | | 7271 | | | 2017-02-15 | | | 2017 | | | Human papillomavirus | | | Unclassified | | | 16-Jul | | | Czechoslovakia | | | Dysk6 | | | skin swab | | | KX781285.1 | | | 1 | | | 0.000212 | | | 0.999788 | | | KX781285.1 | | | 0 | | | 1 | | | 9.18E-12 | | |  |
| MW311489.1 | | 7186 | | | 2022-04-18 | | | 2022 | | | Human papillomavirus | | | Unclassified | | | 16-Nov | | | China | | | Homo sapiens | | |  | | | MW311489.1 | | | 1 | | | 2.55E-07 | | | 1 | | | MW311489.1 | | | 0 | | | 1 | | | 5.41E-10 | | |  |
| KX781286.1 | | 7427 | | | 2017-02-15 | | | 2017 | | | Human papillomavirus | | | Unclassified | | | 16-Jul | | | Czechoslovakia | | | DyskB | | | skin swab | | | KX781286.1 | | | 1 | | | 4.83E-07 | | | 1 | | | KX781286.1 | | | 1 | | | 0.384251 | | | 0.615749 | | |  |
| KX781282.1 | | 7314 | | | 2017-02-15 | | | 2017 | | | Human papillomavirus | | | Unclassified | | | 16-Jul | | | Czechoslovakia | | | Dysk3 | | | skin swab | | | KX781282.1 | | | 1 | | | 0.00041 | | | 0.99959 | | | KX781282.1 | | | 0 | | | 1 | | | 5.01E-08 | | |  |
| JF966379.1 | 7095 | | | 2012-01-11 | | | 2012 | | | Human papillomavirus | | | Unclassified | | | HPV | | | 9-May | | France | | | Homo sapiens | | | 915 F 06 008 CG3 | | | healthy face skin | | | JF966379.1 | | 1 | | 0.002912 | | | 0.997088 | | JF966379.1 | | | 0 | | | 0.992675 | | | 0.007325 | | |
| JF966376.1 | 7219 | | | 2012-01-11 | | | 2012 | | | Human papillomavirus | | | Unclassified | | | HPV | | | 9-May | | France | | | Homo sapiens | | | 915 F 06 007 FD2 | | | healthy face skin | | | JF966376.1 | | 1 | | 0.000188 | | | 0.999812 | | JF966376.1 | | | 0 | | | 0.98358 | | | 0.01642 | | |
| JF966377.1 | 7265 | | | 2012-01-11 | | | 2012 | | | Human papillomavirus | | | Unclassified | | | HPV | | | 9-May | | France | | | Homo sapiens | | | 915 F 06 008 CG1 | | | healthy face skin | | | JF966377.1 | | 1 | | 0.000209 | | | 0.999791 | | JF966377.1 | | | 0 | | | 0.995806 | | | 0.004194 | | |
| OP971101.1 | 7186 | | | 2023-01-14 | | | 2023 | | | human papillomavirus 215 | | | Unclassified | | | HPV 215 | | | ####### | | South Africa | | | Homo sapiens | | | UC145_LW_V1 | | | Vaginal secretion | | | OP971101.1 | | 1 | | 6.81E-11 | | | 1 | | OP971101.1 | | | 0 | | | 1 | | | 9.87E-08 | | |
| OL854075.1 | 7994 | | | 2022-11-15 | | | 2022 | | | Human papillomavirus | | | Unclassified | | | HPV | | | 1-Nov-21 | | China: ShanDong, Jinan | | | Homo sapiens | | | 2133 | | | pathological tissue | | | OL854075.1 | | 0 | | 1 | | | 5.93E-13 | | OL854075.1 | | | 0 | | | 1 | | | 2.30E-08 | | |
| KX781288.1 | | 7328 | | | 2017-02-15 | | | 2017 | | | Human papillomavirus | | | Unclassified | | | 16-Jul | | | Czechoslovakia | | | DyskD | | | skin swab | | | KX781288.1 | | | 1 | | | 1.79E-08 | | | 1 | | | KX781288.1 | | | 0 | | | 0.999891 | | | 0.000109 | | |  |
| MF509821.1 | 7233 | | | 2018-04-13 | | | 2018 | | | Human papillomavirus type 216 | | | Unclassified | | | HPV 216 | | | 2007 | | South Africa | | | Homo sapiens | | | CT12_N | | | penile swab from a south african HIV positive male | | | MF509821.1 | | 0 | | 0.995573 | | | 0.004427 | | MF509821.1 | | | 0 | | | 1 | | | 8.38E-13 | | |
| KP692117.1 | 7320 | | | 2015-08-19 | | | 2015 | | | Human papillomavirus | | | Unclassified | | | HPV | | | 2014 | | Sweden | | | Homo sapiens | | | SE379 | | |  | | | KP692117.1 | | 1 | | 7.65E-05 | | | 0.999923 | | KP692117.1 | | | 0 | | | 0.868107 | | | 0.131893 | | |
| JF966373.1 | 7167 | | | 2012-01-11 | | | 2012 | | | Human papillomavirus | | | Unclassified | | | HPV | | | 9-May | | France | | | Homo sapiens | | | 915 F 06 005 FS1 | | | healthy face skin | | | JF966373.1 | | 1 | | 3.35E-09 | | | 1 | | JF966373.1 | | | 0 | | | 1 | | | 4.26E-08 | | |
| MH172377.1 | 7381 | | | 2018-05-20 | | | 2018 | | | Human papillomavirus type 220 | | | Unclassified | | | HPV 220 | | | 2007 | | South Africa | | | Homo sapiens | | | CT08 | | | penile swab from a South African HIV positive male | | | MH172377.1 | | 1 | | 6.98E-10 | | | 1 | | MH172377.1 | | | 0 | | | 1 | | | 6.92E-08 | | |
| MG063749.1 | 7223 | | | 2018-03-21 | | | 2018 | | | Human papillomavirus | | | Unclassified | | | HPV | | | 1-Jan-17 | | France | | | Homo sapiens | | | MTS3 | | | eyebrows | | | MG063749.1 | | 1 | | 1.56E-05 | | | 0.999984 | | MG063749.1 | | | 0 | | | 0.999722 | | | 0.000278 | | |
| ON482334.1 | 7277 | | | 2023-02-15 | | | 2023 | | | Human papillomavirus 228 | | | Unclassified | | | HPV 228 | | | 2016 | | Mexico | | | Homo sapiens | | | HPV/MEX/0816-030/2016 | | | oral lavage | | | ON482334.1 | | 1 | | 0.241091 | | | 0.758909 | | ON482334.1 | | | 1 | | | 0.001373 | | | 0.998627 | | |
| JF966378.1 | 7152 | | | 2012-01-11 | | | 2012 | | | Human papillomavirus | | | Unclassified | | | HPV | | | 9-May | | France | | | Homo sapiens | | | 915 F 06 008 CG2 | | | healthy face skin | | | JF966378.1 | | 1 | | 3.96E-10 | | | 1 | | JF966378.1 | | | 0 | | | 0.999998 | | | 1.70E-06 | | |
| KF791917.1 | 7190 | | | 2014-01-28 | | | 2014 | | | Human papillomavirus | | | Unclassified | | | HPV | | | 1-Jul-12 | | China | | | Homo sapiens; child | | | HPV-CH2 | | |  | | | KF791917.1 | | 1 | | 0.000184 | | | 0.999816 | | KF791917.1 | | | 1 | | | 0.039981 | | | 0.960019 | | |
| KX781280.1 | | 7335 | | | 2017-02-15 | | | 2017 | | | Human papillomavirus | | | Unclassified | | | 16-Jul | | | Czechoslovakia | | | Dysk1 | | | skin swab | | | KX781280.1 | | | 0 | | | 0.992795 | | | 0.007205 | | | KX781280.1 | | | 0 | | | 1 | | | 4.76E-08 | | |  |
| MH777334.1 | 7288 | | | 2019-01-16 | | | 2019 | | | Human papillomavirus (human skin metagenome) | | | Unclassified | | | HPV | | | 15-Jan | | USA | | | Homo sapiens | | | HPV-mSK_192 | | | skin swab | | | MH777334.1 | | 1 | | 3.09E-05 | | | 0.999969 | | MH777334.1 | | | 0 | | | 1 | | | 9.64E-10 | | |
| MH777330.1 | 7326 | | | 2019-01-16 | | | 2019 | | | Human papillomavirus (human skin metagenome) | | | Unclassified | | | HPV | | | 15-Jan | | USA | | | Homo sapiens | | | HPV-mSK_188 | | | skin swab | | | MH777330.1 | | 0 | | 0.999166 | | | 0.000834 | | MH777330.1 | | | 0 | | | 1 | | | 4.35E-10 | | |
| OP970967.1 | 7299 | | | 2023-01-08 | | | 2023 | | | human papillomavirus mSD2 (human vaginal metagenome) | | | Unclassified | | | HPV mSD2 | | | ####### | | South Africa | | | Homo sapiens | | | UC094_LW_V3 | | | Vaginal secretion | | | OP970967.1 | | 0 | | 0.999818 | | | 0.000182 | | OP970967.1 | | | 0 | | | 0.999869 | | | 0.000131 | | |
| MW679002.1 | 7300 | | | 2021-07-11 | | | 2021 | | | Human papillomavirus AZ1_1 (human nasopharyngeal metagenome) | | | Unclassified | | | HPV AZ1_1 | | | 6-Feb-20 | | USA: Arizona, Tempe | | | Homo sapiens | | | AZ1_1 | | | nasal pharyngeal swab | | | MW679002.1 | | 0 | | 0.999994 | | | 5.64E-06 | | MW679002.1 | | | 0 | | | 0.997728 | | | 0.002272 | | |
| seqID | seqlen | | | date | | | year | | | source | | | type | | | organism | | | collect_date | | country | | | host | | | isolate | | | isolation_source | | | Accession_E7 | | pred_label_E7 | | Score_0_E7 | | | Score_1_E7 | | Accession_E6 | | | pred_label_E6 | | | Score_0_E6 | | | Score_1_E6 | | |
| MF176072.1 | 7216 | | | 2017-10-16 | | | 2017 | | | Human papillomavirus | | | Unclassified | | | HPV | | | ####### | | China | | | Homo sapiens | | | TG550 | | |  | | | MF176072.1 | | 1 | | 6.12E-10 | | | 1 | | MF176072.1 | | | 0 | | | 1 | | | 4.54E-08 | | |
| KY349817.1 | 7405 | | | 2017-09-21 | | | 2017 | | | Human papillomavirus | | | Unclassified | | | HPV | | | 28-Jul-14 | | USA | | | Homo sapiens | | | MTS1 | | | skin | | | KY349817.1 | | 1 | | 8.59E-11 | | | 1 | | KY349817.1 | | | 1 | | | 0.059663 | | | 0.940338 | | |
| KX781281.1 | | 7330 | | | 2017-02-15 | | | 2017 | | | Human papillomavirus | | | Unclassified | | | 16-Jul | | | Czechoslovakia | | | Dysk2 | | | skin swab | | | KX781281.1 | | | 1 | | | 2.80E-07 | | | 1 | | | KX781281.1 | | | 0 | | | 1 | | | 1.95E-07 | | |  |
| MF509818.1 | 7096 | | | 2018-04-13 | | | 2018 | | | Human papillomavirus type 213 | | | Unclassified | | | HPV 213 | | | 2007 | | South Africa | | | Homo sapiens | | | CT04_15 | | | penile swab from a south african HIV negative male | | | MF509818.1 | | 1 | | 1.60E-07 | | | 1 | | MF509818.1 | | | 0 | | | 0.996412 | | | 0.003588 | | |
| MG869605.1 | | | 7240 | | | 2018-09-10 | | | 2018 | | | Human papillomavirus | | | Unclassified | | | Denmark | | | | Homo sapiens | | | oral cavity tumor | | | MG869605.1 | | | 1 | | | 0.066173 | | 0.933827 | | | MG869605.1 | | | | 0 | | | 0.995088 | | | 0.004912 | | |  |  |
| MH172378.1 | 7326 | | | 2018-05-20 | | | 2018 | | | Human papillomavirus type 221 | | | Unclassified | | | HPV 221 | | | 2007 | | South Africa | | | Homo sapiens | | | CT09 | | | penile swab from a South African HIV positive male | | | MH172378.1 | | 1 | | 4.27E-10 | | | 1 | | MH172378.1 | | | 0 | | | 0.999975 | | | 2.52E-05 | | |
| JF966375.1 | 7286 | | | 2012-01-11 | | | 2012 | | | Human papillomavirus | | | Unclassified | | | HPV | | | 9-May | | France | | | Homo sapiens | | | 915 F 06 007 FD1 | | | healthy face skin | | | JF966375.1 | | 0 | | 0.999958 | | | 4.20E-05 | | JF966375.1 | | | 0 | | | 1 | | | 1.44E-11 | | |
| KC113191.1 | | 7299 | | | 2013-04-04 | | | 2013 | | | Human papillomavirus | | | Unclassified | | | 2008 | | | USA | | | Homo sapiens | | | HPV_SD2R.align1 | | | KC113191.1 | | | 0 | | | 0.999302 | | | 0.000698 | | | KC113191.1 | | | 0 | | | 0.999991 | | | 8.75E-06 | | |  |
| KC311731.2 | | 7247 | | | 2013-09-03 | | | 2013 | | | Human papillomavirus | | | Unclassified | | | 2008 | | | Finland | | | Homo sapiens | | | nasal swab | | | KC311731.2 | | | 0 | | | 0.99618 | | | 0.00382 | | | KC311731.2 | | | 0 | | | 1 | | | 4.51E-09 | | |  |
| JF966371.1 | 7300 | | | 2012-01-11 | | | 2012 | | | Human papillomavirus | | | Unclassified | | | HPV | | | 9-May | | France | | | Homo sapiens | | | 915 F 06 002 KN1 | | | healthy face skin | | | JF966371.1 | | 1 | | 0.00023 | | | 0.99977 | | JF966371.1 | | | 0 | | | 1 | | | 5.64E-09 | | |
| MF356498.1 | 7233 | | | 2017-09-21 | | | 2017 | | | Human papillomavirus | | | Unclassified | | | HPV | | | 28-Jul-14 | | USA | | | Homo sapiens | | | ICB1 | | | skin | | | MF356498.1 | | 1 | | 3.98E-07 | | | 1 | | MF356498.1 | | | 0 | | | 0.999996 | | | 3.82E-06 | | |
| JF966372.1 | 7299 | | | 2012-01-11 | | | 2012 | | | Human papillomavirus | | | Unclassified | | | HPV | | | 9-May | | France | | | Homo sapiens | | | 915 F 06 002 KN2 | | | healthy face skin | | | JF966372.1 | | 1 | | 0.290397 | | | 0.709603 | | JF966372.1 | | | 0 | | | 1 | | | 6.16E-12 | | |
| OL854080.1 | 7994 | | | 2022-11-15 | | | 2022 | | | Human papillomavirus | | | Unclassified | | | HPV | | | 1-Nov-21 | | China: ShanDong, Jinan | | | Homo sapiens | | | 1098 | | | pathological tissue | | | OL854080.1 | | 0 | | 1 | | | 5.93E-13 | | OL854080.1 | | | 0 | | | 1 | | | 2.30E-08 | | |
| MK080568.2 | 7441 | | | 2020-02-11 | | | 2020 | | | Human papillomavirus | | | Unclassified | | | HPV | | | ####### | | USA | | | Homo sapiens | | | ICB2 | | | skin | | | MK080568.2 | | 1 | | 5.51E-06 | | | 0.999995 | | MK080568.2 | | | 0 | | | 0.999987 | | | 1.26E-05 | | |
| NC_034616.1 | | 7812 | | | 2018-08-25 | | | 2018 | | | Human papillomavirus type 85 | | | Unclassified | | | Brazil | | | Homo sapiens | | | 114B | | |  | | | NC_034616.1 | | | 1 | | | 0.00098 | | | 0.99902 | | | NC_034616.1 | | | 1 | | | 4.56E-11 | | | 1 | | |  |
| MK645901.1 | 7283 | | | 2019-07-08 | | | 2019 | | | Human papillomavirus | | | Unclassified | | | HPV | | | ####### | | Spain | | | Homo sapiens | | | type 208 | | | oral mucosa | | | MK645901.1 | | 0 | | 0.989719 | | | 0.010281 | | MK645901.1 | | | 0 | | | 1 | | | 3.67E-11 | | |
| KF482069.1 | 7177 | | | 2013-10-24 | | | 2013 | | | Human papillomavirus | | | Unclassified | | | HPV | | | 2013 | | China | | | Homo sapiens | | | HPV-L55 | | | child | | | KF482069.1 | | 1 | | 0.143276 | | | 0.856724 | | KF482069.1 | | | 1 | | | 0.000368 | | | 0.999632 | | |
| MK645900.1 | 7247 | | | 2019-07-08 | | | 2019 | | | Human papillomavirus | | | Unclassified | | | HPV | | | ####### | | Spain | | | Homo sapiens | | | type 207 | | | oral mucosa | | | MK645900.1 | | 0 | | 0.909942 | | | 0.090058 | | MK645900.1 | | | 0 | | | 0.9133 | | | 0.0867 | | |
| KP692119.1 | 7292 | | | 2015-08-19 | | | 2015 | | | Human papillomavirus | | | Unclassified | | | HPV | | | 2014 | | Sweden | | | Homo sapiens | | | SE435 | | |  | | | KP692119.1 | | 1 | | 4.18E-12 | | | 1 | | KP692119.1 | | | 0 | | | 0.999999 | | | 1.17E-06 | | |
| KX781284.1 | | 7281 | | | 2017-02-15 | | | 2017 | | | Human papillomavirus | | | Unclassified | | | 16-Jul | | | Czechoslovakia | | | Dysk5 | | | skin swab | | | KX781284.1 | | | 1 | | | 0.190101 | | | 0.809899 | | | KX781284.1 | | | 0 | | | 1 | | | 2.43E-16 | | |  |
| KX781287.1 | | 7375 | | | 2017-02-15 | | | 2017 | | | Human papillomavirus | | | Unclassified | | | 16-Jul | | | Czechoslovakia | | | DyskC | | | skin swab | | | KX781287.1 | | | 1 | | | 3.26E-09 | | | 1 | | | KX781287.1 | | | 1 | | | 0.347672 | | | 0.652328 | | |  |
| OP971056.1 | 7357 | | | 2023-01-14 | | | 2023 | | | human papillomavirus 214 | | | Unclassified | | | HPV 214 | | | 2-Mar-17 | | South Africa | | | Homo sapiens | | | UC113_LW_V2 | | | Vaginal secretion | | | OP971056.1 | | 0 | | 0.984311 | | | 0.015689 | | OP971056.1 | | | 0 | | | 1 | | | 4.96E-17 | | |
| KJ194499.1 | | 7705 | | | 2014-04-08 | | | 2014 | | | Human papillomavirus | | | Unclassified | | | ####### | | | Netherlands | | | Homo sapiens | | | HPV_Amsterdam_1995 | | | KJ194499.1 | | | 1 | | | 8.76E-12 | | | 1 | | | KJ194499.1 | | | 1 | | | 6.02E-09 | | | 1 | | |  |
| KX514426.1 | | 7812 | | | 2017-05-18 | | | 2017 | | | Human papillomavirus type 85 | | | Unclassified | | | Brazil | | | Homo sapiens | | | 114B | | |  | | | KX514426.1 | | | 1 | | | 0.00098 | | | 0.99902 | | | KX514426.1 | | | 1 | | | 4.56E-11 | | | 1 | | |  |
| KP692118.1 | 7317 | | | 2015-08-19 | | | 2015 | | | Human papillomavirus | | | Unclassified | | | HPV | | | 2014 | | Sweden | | | Homo sapiens | | | SE383 | | |  | | | KP692118.1 | | 1 | | 4.18E-12 | | | 1 | | KP692118.1 | | | 0 | | | 0.999999 | | | 1.17E-06 | | |
| NC_040640.1 | 7372 | | | 2019-09-12 | | | 2019 | | | Human papillomavirus type 203 | | | Unclassified | | | HPV 203 | | | 2014 | | Sweden | | |  | | | SE369 | | | blank paraffin block | | | NC_040640.1 | | 1 | | 0.285294 | | | 0.714706 | | NC_040640.1 | | | 0 | | | 1 | | | 4.32E-09 | | |
| MF509820.1 | 7186 | | | 2018-04-13 | | | 2018 | | | Human papillomavirus type 215 | | | Unclassified | | | HPV 215 | | | 2007 | | South Africa | | | Homo sapiens | | | CT07_2 | | | penile swab from a south african HIV negative male | | | MF509820.1 | | 1 | | 1.28E-11 | | | 1 | | MF509820.1 | | | 0 | | | 1 | | | 2.85E-07 | | |
| MF509817.1 | 7208 | | | 2018-04-13 | | | 2018 | | | Human papillomavirus type 212 | | | Unclassified | | | HPV 212 | | | 2007 | | South Africa | | | Homo sapiens | | | CT03_1 | | | penile swab from a south african HIV positive male | | | MF509817.1 | | 1 | | 0.001683 | | | 0.998317 | | MF509817.1 | | | 0 | | | 0.997821 | | | 0.002179 | | |
| MG921180.1 | 7372 | | | 2018-06-05 | | | 2018 | | | Human papillomavirus type 203 | | | Unclassified | | | HPV 203 | | | 2014 | | Sweden | | |  | | | SE369 | | | blank paraffin block | | | MG921180.1 | | 1 | | 0.285294 | | | 0.714706 | | MG921180.1 | | | 0 | | | 1 | | | 4.32E-09 | | |
| MH172379.1 | 7275 | | | 2018-05-20 | | | 2018 | | | Human papillomavirus type 222 | | | Unclassified | | | HPV 222 | | | 2007 | | South Africa | | | Homo sapiens | | | CT17 | | | penile swab from a South African HIV negative male | | | MH172379.1 | | 1 | | 1.22E-12 | | | 1 | | MH172379.1 | | | 0 | | | 1 | | | 3.36E-10 | | |
| MG520499.1 | 7320 | | | 2018-03-21 | | | 2018 | | | Human papillomavirus | | | Unclassified | | | HPV | | | 1-Jan-17 | | France | | | Homo sapiens | | | MTS4 | | | eyebrows | | | MG520499.1 | | 1 | | 0.000108 | | | 0.999892 | | MG520499.1 | | | 0 | | | 1 | | | 3.55E-09 | | |
| OL854078.1 | 7994 | | | 2022-11-15 | | | 2022 | | | Human papillomavirus | | | Unclassified | | | HPV | | | 1-Nov-21 | | China: ShanDong, Jinan | | | Homo sapiens | | | 1122 | | | pathological tissue | | | OL854078.1 | | 0 | | 1 | | | 5.93E-13 | | OL854078.1 | | | 0 | | | 1 | | | 2.30E-08 | | |
| OL854079.1 | 7994 | | | 2022-11-15 | | | 2022 | | | Human papillomavirus | | | Unclassified | | | HPV | | | 1-Nov-21 | | China: ShanDong, Jinan | | | Homo sapiens | | | 723 | | | pathological tissue | | | OL854079.1 | | 0 | | 1 | | | 5.93E-13 | | OL854079.1 | | | 0 | | | 1 | | | 2.30E-08 | | |
| MF509816.1 | 7253 | | | 2018-04-13 | | | 2018 | | | Human papillomavirus type 211 | | | Unclassified | | | HPV 211 | | | 2007 | | South Africa | | | Homo sapiens | | | CT02_8 | | | penile swab from a south african HIV negative male | | | MF509816.1 | | 1 | | 4.27E-09 | | | 1 | | MF509816.1 | | | 0 | | | 0.999946 | | | 5.36E-05 | | |
| OL854076.1 | 7994 | | | 2022-11-15 | | | 2022 | | | Human papillomavirus | | | Unclassified | | | HPV | | | 1-Nov-21 | | China: ShanDong, Jinan | | | Homo sapiens | | | 2004 | | | pathological tissue | | | OL854076.1 | | 0 | | 1 | | | 5.93E-13 | | OL854076.1 | | | 0 | | | 1 | | | 2.30E-08 | | |
| OL854077.1 | 7994 | | | 2022-11-15 | | | 2022 | | | Human papillomavirus | | | Unclassified | | | HPV | | | 1-Nov-21 | | China: ShanDong, Jinan | | | Homo sapiens | | | 1162 | | | pathological tissue | | | OL854077.1 | | 0 | | 1 | | | 5.93E-13 | | OL854077.1 | | | 0 | | | 1 | | | 2.30E-08 | | |
| LR862055.1 | | | 7096 | | | 2020-09-01 | | | 2020 | | | Human papillomavirus type 213 | | | Unclassified | | | Luxembourg | | | | Homo sapiens | | | LNS8643648_HPV213 | | | LR862055.1 | | | 1 | | | 1.60E-07 | | 1 | | | LR862055.1 | | | | 0 | | | 0.997563 | | | 0.002437 | | |  |  |
| MG869604.1 | | 7291 | | | 2018-09-10 | | | 2018 | | | Human papillomavirus | | | Unclassified | | | ####### | | | Denmark | | | Homo sapiens | | | mycosis fungoides tumor | | | MG869604.1 | | | 1 | | | 5.83E-06 | | | 0.999994 | | | MG869604.1 | | | 0 | | | 0.999929 | | | 7.15E-05 | | |  |
| KX781283.1 | | 7306 | | | 2017-02-15 | | | 2017 | | | Human papillomavirus | | | Unclassified | | | 16-Jul | | | Czechoslovakia | | | Dysk4 | | | skin swab | | | KX781283.1 | | | 1 | | | 0.001862 | | | 0.998138 | | | KX781283.1 | | | 0 | | | 1 | | | 2.55E-07 | | |  |
| JF966374.1 | 7251 | | | 2012-01-11 | | | 2012 | | | Human papillomavirus | | | Unclassified | | | HPV | | | 9-May | | France | | | Homo sapiens | | | 915 F 06 002 KN3 | | | healthy face skin | | | JF966374.1 | | 1 | | 0.017846 | | | 0.982155 | | JF966374.1 | | | 0 | | | 1 | | | 2.62E-16 | | |
| KY780961.1 | 7319 | | | 2017-09-21 | | | 2017 | | | Human papillomavirus | | | Unclassified | | | HPV | | | 29-Jul-14 | | USA | | | Homo sapiens | | | MTS2 | | | skin | | | KY780961.1 | | 0 | | 0.814786 | | | 0.185214 | | KY780961.1 | | | 0 | | | 1 | | | 7.91E-08 | | |
| NC_027779.1 | 7320 | | | 2018-08-13 | | | 2018 | | | Human papillomavirus | | | Unclassified | | | HPV | | | 2014 | | Sweden | | | Homo sapiens | | | SE379 | | |  | | | NC_027779.1 | | 1 | | 7.65E-05 | | | 0.999923 | | NC_027779.1 | | | 0 | | | 0.868107 | | | 0.131893 | | |
| MF588757.1 | 7203 | | | 2019-03-22 | | | 2019 | | | Human papillomavirus sp | | | Unclassified | | | HPV sp. | | | 14-May | | Italy | | | Homo sapiens | | | Mu3_d01c06 | | | skin swabs from Dock8 (dedicator of cytokinesis) deficiency, forehead, back, groin | | | MF588757.1 | | 1 | | 0.01065 | | | 0.98935 | | MF588757.1 | | | 0 | | | 0.933794 | | | 0.066206 | | |
| MH172376.1 | 7108 | | | 2018-05-20 | | | 2018 | | | Human papillomavirus type 219 | | | Unclassified | | | HPV 219 | | | 2007 | | South Africa | | | Homo sapiens | | | CT01 | | | penile swab from a South African HIV positive male | | | MH172376.1 | | 1 | | 0.000526 | | | 0.999474 | | MH172376.1 | | | 0 | | | 0.999512 | | | 0.000488 | | |
| KX781285.1 | | 7271 | | | 2017-02-15 | | | 2017 | | | Human papillomavirus | | | Unclassified | | | 16-Jul | | | Czechoslovakia | | | Dysk6 | | | skin swab | | | KX781285.1 | | | 1 | | | 0.000212 | | | 0.999788 | | | KX781285.1 | | | 0 | | | 1 | | | 9.18E-12 | | |  |
| MW311489.1 | | 7186 | | | 2022-04-18 | | | 2022 | | | Human papillomavirus | | | Unclassified | | | 16-Nov | | | China | | | Homo sapiens | | |  | | | MW311489.1 | | | 1 | | | 2.55E-07 | | | 1 | | | MW311489.1 | | | 0 | | | 1 | | | 5.41E-10 | | |  |
| KX781286.1 | | 7427 | | | 2017-02-15 | | | 2017 | | | Human papillomavirus | | | Unclassified | | | 16-Jul | | | Czechoslovakia | | | DyskB | | | skin swab | | | KX781286.1 | | | 1 | | | 4.83E-07 | | | 1 | | | KX781286.1 | | | 1 | | | 0.384251 | | | 0.615749 | | |  |
| KX781282.1 | | 7314 | | | 2017-02-15 | | | 2017 | | | Human papillomavirus | | | Unclassified | | | 16-Jul | | | Czechoslovakia | | | Dysk3 | | | skin swab | | | KX781282.1 | | | 1 | | | 0.00041 | | | 0.99959 | | | KX781282.1 | | | 0 | | | 1 | | | 5.01E-08 | | |  |
| JF966379.1 | 7095 | | | 2012-01-11 | | | 2012 | | | Human papillomavirus | | | Unclassified | | | HPV | | | 9-May | | France | | | Homo sapiens | | | 915 F 06 008 CG3 | | | healthy face skin | | | JF966379.1 | | 1 | | 0.002912 | | | 0.997088 | | JF966379.1 | | | 0 | | | 0.992675 | | | 0.007325 | | |
| JF966376.1 | 7219 | | | 2012-01-11 | | | 2012 | | | Human papillomavirus | | | Unclassified | | | HPV | | | 9-May | | France | | | Homo sapiens | | | 915 F 06 007 FD2 | | | healthy face skin | | | JF966376.1 | | 1 | | 0.000188 | | | 0.999812 | | JF966376.1 | | | 0 | | | 0.98358 | | | 0.01642 | | |
| JF966377.1 | 7265 | | | 2012-01-11 | | | 2012 | | | Human papillomavirus | | | Unclassified | | | HPV | | | 9-May | | France | | | Homo sapiens | | | 915 F 06 008 CG1 | | | healthy face skin | | | JF966377.1 | | 1 | | 0.000209 | | | 0.999791 | | JF966377.1 | | | 0 | | | 0.995806 | | | 0.004194 | | |
| OP971101.1 | 7186 | | | 2023-01-14 | | | 2023 | | | human papillomavirus 215 | | | Unclassified | | | HPV 215 | | | ####### | | South Africa | | | Homo sapiens | | | UC145_LW_V1 | | | Vaginal secretion | | | OP971101.1 | | 1 | | 6.81E-11 | | | 1 | | OP971101.1 | | | 0 | | | 1 | | | 9.87E-08 | | |
| OL854075.1 | 7994 | | | 2022-11-15 | | | 2022 | | | Human papillomavirus | | | Unclassified | | | HPV | | | 1-Nov-21 | | China: ShanDong, Jinan | | | Homo sapiens | | | 2133 | | | pathological tissue | | | OL854075.1 | | 0 | | 1 | | | 5.93E-13 | | OL854075.1 | | | 0 | | | 1 | | | 2.30E-08 | | |
| KX781288.1 | | 7328 | | | 2017-02-15 | | | 2017 | | | Human papillomavirus | | | Unclassified | | | 16-Jul | | | Czechoslovakia | | | DyskD | | | skin swab | | | KX781288.1 | | | 1 | | | 1.79E-08 | | | 1 | | | KX781288.1 | | | 0 | | | 0.999891 | | | 0.000109 | | |  |
| MF509821.1 | 7233 | | | 2018-04-13 | | | 2018 | | | Human papillomavirus type 216 | | | Unclassified | | | HPV 216 | | | 2007 | | South Africa | | | Homo sapiens | | | CT12_N | | | penile swab from a south african HIV positive male | | | MF509821.1 | | 0 | | 0.995573 | | | 0.004427 | | MF509821.1 | | | 0 | | | 1 | | | 8.38E-13 | | |
| KP692117.1 | 7320 | | | 2015-08-19 | | | 2015 | | | Human papillomavirus | | | Unclassified | | | HPV | | | 2014 | | Sweden | | | Homo sapiens | | | SE379 | | |  | | | KP692117.1 | | 1 | | 7.65E-05 | | | 0.999923 | | KP692117.1 | | | 0 | | | 0.868107 | | | 0.131893 | | |
| JF966373.1 | 7167 | | | 2012-01-11 | | | 2012 | | | Human papillomavirus | | | Unclassified | | | HPV | | | 9-May | | France | | | Homo sapiens | | | 915 F 06 005 FS1 | | | healthy face skin | | | JF966373.1 | | 1 | | 3.35E-09 | | | 1 | | JF966373.1 | | | 0 | | | 1 | | | 4.26E-08 | | |
| MH172377.1 | 7381 | | | 2018-05-20 | | | 2018 | | | Human papillomavirus type 220 | | | Unclassified | | | HPV 220 | | | 2007 | | South Africa | | | Homo sapiens | | | CT08 | | | penile swab from a South African HIV positive male | | | MH172377.1 | | 1 | | 6.98E-10 | | | 1 | | MH172377.1 | | | 0 | | | 1 | | | 6.92E-08 | | |
| MG063749.1 | 7223 | | | 2018-03-21 | | | 2018 | | | Human papillomavirus | | | Unclassified | | | HPV | | | 1-Jan-17 | | France | | | Homo sapiens | | | MTS3 | | | eyebrows | | | MG063749.1 | | 1 | | 1.56E-05 | | | 0.999984 | | MG063749.1 | | | 0 | | | 0.999722 | | | 0.000278 | | |
| ON482334.1 | 7277 | | | 2023-02-15 | | | 2023 | | | Human papillomavirus 228 | | | Unclassified | | | HPV 228 | | | 2016 | | Mexico | | | Homo sapiens | | | HPV/MEX/0816-030/2016 | | | oral lavage | | | ON482334.1 | | 1 | | 0.241091 | | | 0.758909 | | ON482334.1 | | | 1 | | | 0.001373 | | | 0.998627 | | |
| JF966378.1 | 7152 | | | 2012-01-11 | | | 2012 | | | Human papillomavirus | | | Unclassified | | | HPV | | | 9-May | | France | | | Homo sapiens | | | 915 F 06 008 CG2 | | | healthy face skin | | | JF966378.1 | | 1 | | 3.96E-10 | | | 1 | | JF966378.1 | | | 0 | | | 0.999998 | | | 1.70E-06 | | |
| KF791917.1 | 7190 | | | 2014-01-28 | | | 2014 | | | Human papillomavirus | | | Unclassified | | | HPV | | | 1-Jul-12 | | China | | | Homo sapiens; child | | | HPV-CH2 | | |  | | | KF791917.1 | | 1 | | 0.000184 | | | 0.999816 | | KF791917.1 | | | 1 | | | 0.039981 | | | 0.960019 | | |
| KX781280.1 | | 7335 | | | 2017-02-15 | | | 2017 | | | Human papillomavirus | | | Unclassified | | | 16-Jul | | | Czechoslovakia | | | Dysk1 | | | skin swab | | | KX781280.1 | | | 0 | | | 0.992795 | | | 0.007205 | | | KX781280.1 | | | 0 | | | 1 | | | 4.76E-08 | | |  |
| MH777334.1 | 7288 | | | 2019-01-16 | | | 2019 | | | Human papillomavirus (human skin metagenome) | | | Unclassified | | | HPV | | | 15-Jan | | USA | | | Homo sapiens | | | HPV-mSK_192 | | | skin swab | | | MH777334.1 | | 1 | | 3.09E-05 | | | 0.999969 | | MH777334.1 | | | 0 | | | 1 | | | 9.64E-10 | | |
| MH777330.1 | 7326 | | | 2019-01-16 | | | 2019 | | | Human papillomavirus (human skin metagenome) | | | Unclassified | | | HPV | | | 15-Jan | | USA | | | Homo sapiens | | | HPV-mSK_188 | | | skin swab | | | MH777330.1 | | 0 | | 0.999166 | | | 0.000834 | | MH777330.1 | | | 0 | | | 1 | | | 4.35E-10 | | |
| OP970967.1 | 7299 | | | 2023-01-08 | | | 2023 | | | human papillomavirus mSD2 (human vaginal metagenome) | | | Unclassified | | | HPV mSD2 | | | ####### | | South Africa | | | Homo sapiens | | | UC094_LW_V3 | | | Vaginal secretion | | | OP970967.1 | | 0 | | 0.999818 | | | 0.000182 | | OP970967.1 | | | 0 | | | 0.999869 | | | 0.000131 | | |
| MW679002.1 | 7300 | | | 2021-07-11 | | | 2021 | | | Human papillomavirus AZ1_1 (human nasopharyngeal metagenome) | | | Unclassified | | | HPV AZ1_1 | | | 6-Feb-20 | | USA: Arizona, Tempe | | | Homo sapiens | | | AZ1_1 | | | nasal pharyngeal swab | | | MW679002.1 | | 0 | | 0.999994 | | | 5.64E-06 | | MW679002.1 | | | 0 | | | 0.997728 | | | 0.002272 | | |
